# Zinc Depletion Increases Susceptibility to AMPK-Induced Atrophic Responses in C2C12 Myotubes

**DOI:** 10.3390/pathophysiology33010012

**Published:** 2026-02-02

**Authors:** Taishi Imoto, Junpei Ishizaka, Yukinori Tamura

**Affiliations:** Division of Physiology and Biochemistry, Faculty of Nutrition, Kobe Gakuin University, 518 Arise, Ikawadani-cho, Nishi-ku, Kobe 651-2180, Japan

**Keywords:** AMP-activated protein kinase, zinc depletion, skeletal muscle atrophy, ubiquitin–proteasome system, FoxO transcription factors

## Abstract

**Background**: AMP-activated protein kinase (AMPK) acts as a key energy sensor that negatively regulates skeletal muscle mass. Zinc is an essential trace element that is required for myogenic differentiation and protein synthesis, while zinc deficiency has been associated with muscle atrophy in vivo. However, how zinc status modulates AMPK activation itself or alters downstream responses to AMPK signaling in muscle cells remains unclear. **Methods**: C2C12 myotubes were cultured under zinc-depleted (ZnD), zinc-sufficient (20 μM; Zn20), or zinc-supplemented (40 μM; Zn40) conditions. AMPK was activated by AICAR, and zinc status–dependent responses were evaluated using molecular and morphological analyses. **Results**: AICAR increased intracellular zinc levels in Zn20 and Zn40 but not in ZnD. Zinc transporter expression exhibited gene-specific regulation: *Zip3* was upregulated across all zinc conditions, *Zip14* was significantly induced in ZnD and Zn40, and *Zip10* was selectively upregulated in Zn40. AICAR induced myotube atrophy in all groups; however, the reduction in myotube diameter was significantly greater under zinc-depleted conditions. Zinc depletion was associated with transcriptional upregulation of FoxO1, FoxO3, Atrogin-1, and MuRF1 in response to AICAR, while AMPK activation and suppression of S6K1 phosphorylation occurred to a similar extent regardless of zinc status. **Conclusions**: These findings indicate that zinc availability does not alter AMPK activation itself but modulates downstream atrophic responses to AMPK signaling. Under conditions of AMPK activation, adequate zinc availability is accompanied by increased intracellular zinc levels and stress-responsive ZIP regulation, which may limit excessive atrophic gene induction, whereas zinc depletion increases susceptibility to AMPK-induced atrophic responses in skeletal muscle cells.

## 1. Introduction

Skeletal muscle, comprising nearly half of body mass, is essential for locomotion, metabolism, and systemic homeostasis. Muscle mass depends on the balance between protein synthesis and degradation, and disruption of this equilibrium underlies sarcopenia in aging and cachexia in chronic disease [1,2,3].

AMP-activated protein kinase (AMPK) serves as a central regulator of cellular energy balance, activated by metabolic stresses such as fasting, exercise, or hypoxia that increase the AMP/ATP ratio [4]. In skeletal muscle, AMPK suppresses the Akt–mTORC1-S6K1 anabolic signaling while activating catabolic gene programs, including genes associated with the ubiquitin–proteasome system and autophagy, to conserve energy under stress conditions [5]. Both α1 and α2 catalytic subunits expressed in muscle contribute to this catabolic regulation by limiting hypertrophic growth and promoting atrophy-related signaling [6,7]. However, AMPK activation is not uniformly detrimental; pharmacological activation by AICAR can counteract inflammation-induced cachectic muscle wasting [8], and sustained activation enhances oxidative remodeling and fiber regeneration in mitochondrial myopathy models [9]. Furthermore, AMPK supports mitochondrial maintenance and metabolic flexibility, thereby contributing to skeletal muscle health [10]. Thus, AMPK acts as a context-dependent modulator that integrates catabolic and adaptive remodeling pathways to preserve muscle integrity.

Zinc, an essential trace element acting as a structural and catalytic cofactor for hundreds of enzymes, is abundant in skeletal muscle and bone [11,12]. Zinc deficiency remains common worldwide, leading to growth retardation, immune dysfunction, and delayed tissue repair [13]. Previous studies suggest that zinc can promote myoblast proliferation [14] and myogenic differentiation [15]. Conversely, zinc deficiency inhibits protein synthesis [16] and has been associated with mitochondrial dysfunction and muscle atrophy in the skeletal muscle of experimental animals [17].

Despite these findings, the relationship between zinc status and AMPK-associated responses in muscle is poorly defined. Nutritional factors—including vitamins and minerals—are known to influence downstream metabolic programs regulated by AMPK, rather than AMPK activation itself [18,19,20,21]. In addition, zinc availability can influence AMPK-linked lipid metabolism in the liver [22], suggesting that micronutrient status may shape cellular responses to AMPK signaling beyond classical energetic regulation. Because AMPK is activated under metabolic stress and zinc deficiency represents such a stressor, we hypothesized that inadequate zinc availability modifies downstream atrophic responses to AMPK activation, rather than altering AMPK activation per se. This study therefore examined how zinc status affects susceptibility to AMPK-induced atrophic responses, focusing on transcriptional regulation of zinc transporters, FoxO factors, and UPS-related genes in C2C12 myotubes.

## 2. Materials and Methods

### 2.1. Cell Culture and Experimental Design

C2C12 myoblasts (ATCC, Manassas, VA, USA) were cultured in growth medium (DMEM containing 4.5 g/L glucose, 10% fetal bovine serum (Biowest, Nuaillé, France), and 1% penicillin–streptomycin) at 37 °C in a humidified atmosphere of 5% CO_2_. For differentiation, cells were switched to DMEM supplemented with 2% horse serum (Cytiva, Tokyo, Japan) and 1% penicillin–streptomycin for 6 days to form multinucleated myotubes. Zinc-depleted serum was prepared by stirring horse serum overnight with Chelex 100 resin (Bio-Rad, Hercules, CA, USA) to remove divalent cations. As a verification step, zinc concentrations in horse serum were measured before and after Chelex treatment, decreasing from 67.7 μg/dL to 9.7 μg/dL. However, extracellular zinc concentrations in the final complete media were not directly quantified. Zinc-depleted (ZnD) medium served as the base for all zinc conditions, and ZnSO_4_ was added to obtain zinc-sufficient (20 µM; Zn20) and zinc-supplemented (40 µM; Zn40) media. In this study, Zn20 was used as a zinc-sufficient reference condition rather than a strict physiological control, representing a commonly used zinc-replete culture condition for comparative analyses, whereas ZnD and Zn40 were designed as deviations from this baseline to model zinc depletion and zinc supplementation, respectively. This concentration was selected because it approximates the normal range of human plasma zinc concentrations (11.4–17.8 μM), as summarized in a previous review on zinc homeostasis [12]. All experimental groups were prepared using the same Chelex-treated basal medium, with zinc as the only divalent cation selectively repleted by ZnSO_4_ supplementation. Myotubes were preincubated for 3 h in each respective medium, followed by incubation under the same zinc condition with Vehicle (Control) or AICAR (1 mM; FujiFilm Wako, Osaka, Japan) for 24 h to induce AMPK activation.

### 2.2. Measurement of Myotube Diameter

Phase-contrast micrographs were captured using a 20× objective lens. More than 150 myotubes per sample were randomly selected, and their diameters were measured using ImageJ software (version 1.54i; NIH, Bethesda, MD, USA). For each myotube, three distinct points along its length were measured and averaged to obtain a single mean value. Field selection and diameter measurements were performed in a randomized and blinded manner.

### 2.3. Determination of Intracellular Zinc Concentration

Cells were lysed in 250 µL of extraction buffer composed of cOmplete™ Lysis-M, EDTA-free Reagent (Roche Diagnostics KK, Tokyo, Japan) supplemented with 1% phosphatase inhibitor cocktail, 1% protease inhibitor cocktail, and 1 mM PMSF. Zinc concentration in the lysates was quantified using the Metalloassay Zinc LS kit (Metallogenics Co., Ltd., Chiba, Japan) and normalized to total protein concentration determined by the Pierce™ BCA Protein Assay Kit (ThermoFisher Scientific, Waltham, MA, USA).

### 2.4. Real-Time Quantitative PCR

Total RNA was isolated using the RNeasy Mini Kit (Qiagen, Hilden, Germany) and reverse-transcribed using the High-Capacity cDNA Reverse Transcription Kit (Applied Biosystems, Foster City, CA, USA). Real-time PCR was performed on a LightCycler 96 system (Roche Diagnostics KK) using the FastStart Essential DNA Green Master reagent (Roche Diagnostics KK). Thermal cycling conditions were as follows: preincubation at 95 °C for 10 min; amplification for 45 cycles of 95 °C for 10 s, 55 °C for 10 s, and 72 °C for 10 s. Gene expression levels were normalized to Gapdh as an internal reference using the ΔΔCt method. Primer sequences are listed in Supplementary Table S1.

### 2.5. Western Blot Analysis

Cells were lysed in cOmplete™ Lysis-M, EDTA-free Reagent (Roche Diagnostics KK) supplemented with 1% phosphatase inhibitor cocktail, 1% protease inhibitor cocktail, and 1 mM PMSF. Total protein concentration was determined by the BCA assay. Equal amounts of protein (10 µg) were separated by SDS–PAGE using a 4–20% precast gradient gel (Bio-Rad, Hercules, CA, USA) at a constant voltage of 150 V for 40 min. PVDF membranes were activated by brief methanol immersion (prior to transfer), and proteins were then transferred onto the membranes (Merck Millipore, Burlington, MA, USA) at 100 V for 50 min using a wet transfer system. The membranes were blocked in 2% skim milk/TBST for 1 h at room temperature and incubated overnight at 4 °C with primary antibodies diluted 1:1000 in 2% skim milk/TBST. After washing (3 × 10 min, TBST), membranes were incubated with HRP-conjugated anti-rabbit IgG secondary antibody (Cell Signaling Technology, Danvers, MA, USA; Cat. #7074, 1:2000) for 1 h at room temperature. Immunoreactive bands were visualized using Amersham ECL Prime (Cytiva, Tokyo, Japan) and imaged with a LuminoGraph I (ATTO, Tokyo, Japan). Band intensity was quantified using CS Analyzer 4 (ATTO). AMPK activation was assessed by calculating the ratio of phospho-AMPK (Thr172) to total AMPK to account for potential changes in total AMPK protein expression following AICAR treatment. Total AMPK and GAPDH were used to confirm equal protein loading across conditions. The following primary antibodies were used: phospho-AMPK (Thr172; Cat. #2535), total AMPK (Cat. #2532), phospho-p70S6K1 (Thr389; Cat. #9205), total p70S6K1 (Cat. #9202) and GAPDH (Cat. #2118), all purchased from Cell Signaling Technology (Danvers, MA, USA).

### 2.6. Statistical Analysis

Statistical analyses were performed using GraphPad Prism 8 (GraphPad Software, San Diego, CA, USA). Data are presented as means ± SEM. n denotes independent cultures derived from separate differentiation experiments. Differences among groups were analyzed using two-way ANOVA to assess the main effects of zinc condition and AICAR treatment and their interaction, followed by the Holm–Sidak post hoc test for multiple comparisons. Data satisfied normality and homoscedasticity assumptions. *p* < 0.05 was considered statistically significant.

## 3. Results

### 3.1. Zinc Depletion Is Associated with Increased Susceptibility to AMPK-Induced Myotube Atrophy

**Figure 1:**
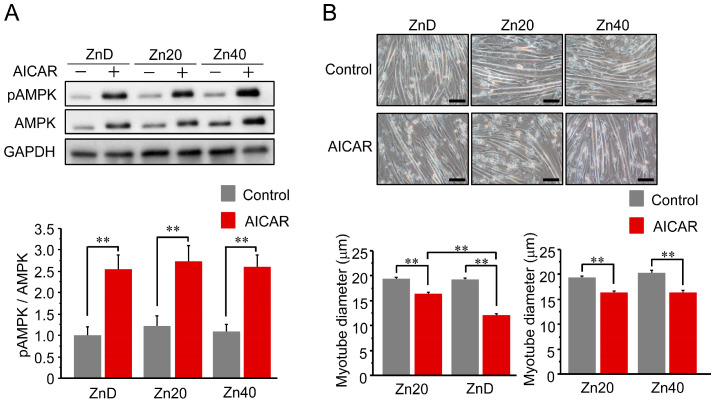
Zinc depletion is associated with increased susceptibility to AMPK-induced myotube atrophy. (**A**) Representative Western blot showing phosphorylated and total AMP-activated protein kinase (pAMPK and AMPK) after AICAR stimulation (1 mM, 24 h) in C2C12 myotubes cultured under zinc-depleted (ZnD), zinc-sufficient (Zn20; 20 μM ZnSO_4_), or zinc-supplemented (Zn40; 40 μM ZnSO_4_) conditions. GAPDH was used as a loading control. (**B**) Representative phase-contrast images of myotubes (scale bar = 100 μm) and quantitative analysis of mean myotube diameter. Data are presented as mean ± SEM (n = 3 in each group). ** *p* < 0.01. Statistical comparisons include Control vs. AICAR within each zinc condition, as well as comparisons among zinc conditions where indicated. Zn20 (20 μM ZnSO_4_) was used as a zinc-sufficient reference condition for comparative analyses in this in vitro system.

AICAR treatment increased AMPK phosphorylation (pAMPK/AMPK ratio) in all groups, and the extent of activation was comparable among ZnD, Zn20, and Zn40 myotubes (Figure 1A). Despite this equivalent activation of AMPK, the reduction in myotube diameter following AICAR treatment was significantly greater in ZnD than in Zn20 and Zn40 (Figure 1B). These findings indicate that zinc depletion increases the susceptibility of myotubes to AMPK-induced atrophy without altering their responsiveness to AMPK activation.

### 3.2. AICAR Enhanced Intracellular Zinc Only in Zinc-Sufficient Myotubes, Accompanied by Differential Expression of Zip Transporters

**Figure 2:**
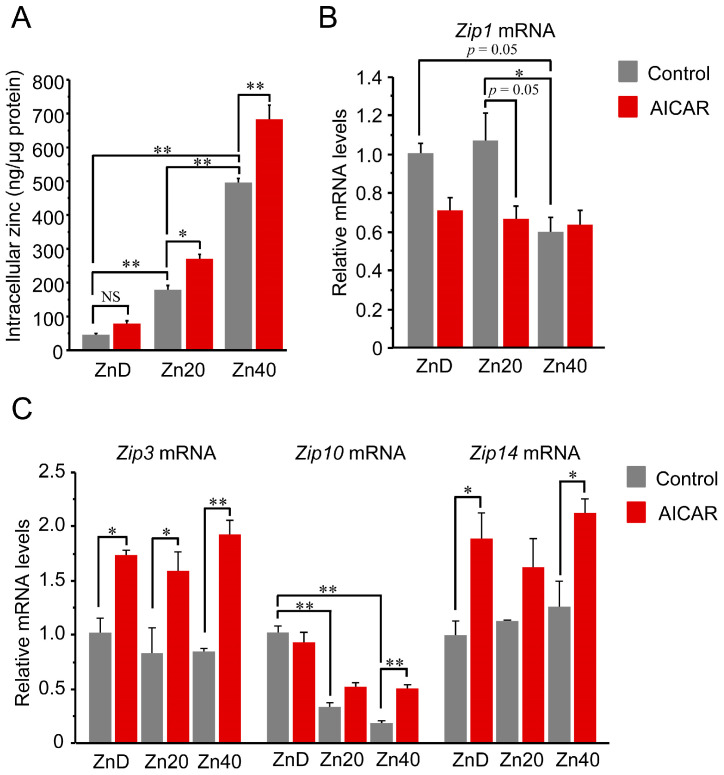
AICAR increased intracellular zinc only in zinc-sufficient and -supplemented myotubes, with distinct ZIP transporter responses. (**A**) Intracellular zinc concentration normalized to total protein content. (**B**,**C**) Relative mRNA expression of zinc transporters *Zip1* (**B**) and *Zip3*, *Zip10*, *Zip14* (**C**) determined by quantitative PCR. Two-way ANOVA revealed significant main effects of AICAR treatment and zinc condition, as well as their interaction, for *Zip1* (*p* < 0.05 for all). *Zip1* expression tended to decrease after AICAR stimulation under ZnD and Zn20 conditions, and was already lower at baseline in Zn40 compared with ZnD and Zn20. For *Zip3*, *Zip14*, and *Zip10*, AICAR main effects were significant (*p* < 0.05 for all). A significant zinc main effect and zinc × AICAR interaction were additionally observed only for *Zip10* (*p* < 0.05). Post hoc comparisons are indicated by asterisks. Data are presented as mean ± SEM (n = 3 per group). * *p* < 0.05, ** *p* < 0.01, NS: not significant. Zn20 (20 μM ZnSO_4_) was used as a zinc-sufficient reference condition for comparative analyses in this in vitro system.

Intracellular zinc content increased in a zinc dose-dependent manner and was further elevated by AICAR in Zn20 and Zn40, but not in ZnD (Figure 2A). Regarding zinc transporters, *Zip1* (*SLC39A1*) tended to decrease after AICAR in ZnD and Zn20, while its basal expression was already lower in Zn40 compared with ZnD and Zn20 (Figure 2B). Two-way ANOVA indicated significant main effects of zinc condition and AICAR, as well as a zinc × AICAR interaction for *Zip1* (*p* < 0.05 for all), supporting that *Zip1* expression is modulated by both zinc status and AMPK activation. In contrast, *Zip3* (*SLC39A3*) showed a significant AICAR-induced increase under all zinc conditions, whereas *Zip14* (*SLC39A14*) exhibited a similar upward trend but reached statistical significance only in ZnD and Zn40, likely due to larger variability in Zn20 (Figure 2C). *Zip10* (*SLC39A10*) exhibited a distinct pattern: its basal expression was higher under ZnD and declined with increasing extracellular zinc; upon AICAR stimulation, *Zip10* remained unchanged in ZnD and Zn20, while significantly increased only in Zn40 (Figure 2C). Two-way ANOVA revealed significant main effects of AICAR for *Zip3*, *Zip14*, and *Zip10* (all *p* < 0.05). A significant main effect of zinc condition and a zinc × AICAR interaction were observed only for *Zip10* (*p* < 0.05). These data indicate that AMPK activation is associated with increased intracellular zinc levels when extracellular zinc is sufficient, involving broad induction of Zip3/ZIP14 and zinc-dependent regulation of ZIP1 and ZIP10.

### 3.3. Zinc Depletion Is Associated with AMPK-Induced Transcriptional Upregulation of UPS-Related Genes with Distinct Regulation of MuRF1 and Atrogin-1 at the mRNA Level

**Figure 3:**
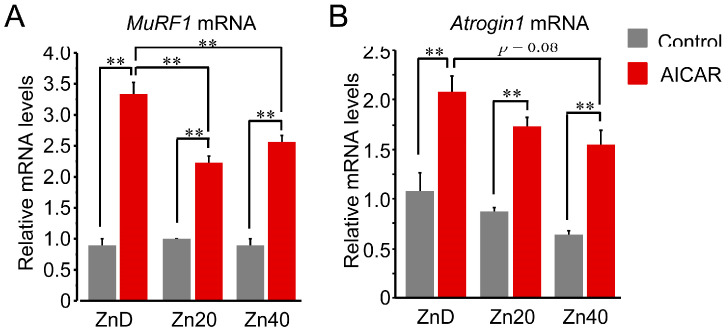
Zinc depletion is associated with AMPK-induced transcriptional upregulation of the ubiquitin-proteasome-related genes with distinct regulation of MuRF1 and Atrogin-1 at the mRNA level. (**A**,**B**) Relative mRNA expression levels of Trim63 (MuRF1) (**A**) and Fbxo32 (Atrogin-1) (**B**) in myotubes cultured under ZnD, Zn20, or Zn40 conditions, with or without AICAR treatment. Data are presented as mean ± SEM (n = 3 in each group). ** *p* < 0.01. Statistical comparisons include Control vs. AICAR within each zinc condition, as well as comparisons among zinc conditions where indicated. Zn20 (20 μM ZnSO_4_) was used as a zinc-sufficient reference condition for comparative analyses in this in vitro system.

At baseline, *Trim63* (*MuRF1*) mRNA expression showed no significant difference among ZnD, Zn20, and Zn40 myotubes (Figure 3A). AICAR stimulation markedly increased *MuRF1* mRNA expression in all groups, but the induction was significantly greater in ZnD and was significantly attenuated in Zn20 and Zn40. In contrast, *Fbxo32* (*Atrogin-1*) mRNA tended to be higher at baseline under ZnD compared with Zn20 and Zn40, and following AICAR stimulation, its expression reached the highest level in ZnD, decreasing progressively with higher zinc concentrations (Figure 3B). These results indicate that zinc depletion augments AMPK-induced transcriptional upregulation of ubiquitin–proteasome–related genes, with *MuRF1* and *Atrogin-1* exhibiting distinct zinc-dependent responses.

### 3.4. Zinc Depletion Is Associated with Greater AMPK-Induced Up-Regulation of FoxO1 and FoxO3 at the mRNA Level

**Figure 4:**
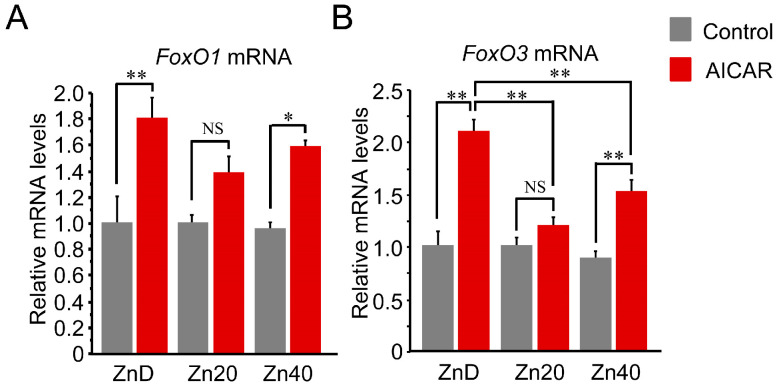
Zinc depletion is associated with greater AMPK-induced transcriptional upregulation of FoxO1 and FoxO3 at the mRNA level. (**A**,**B**) Relative mRNA expression of *FoxO1* (**A**) and *FoxO3* (**B**) in C2C12 myotubes cultured under ZnD, Zn20, or Zn40 conditions, with or without AICAR treatment. Data are presented as mean ± SEM (n = 3 in each group). * *p* < 0.05, ** *p* < 0.01, NS: not significant. Statistical comparisons include Control vs. AICAR within each zinc condition, as well as comparisons among zinc conditions where indicated. Zn20 (20 μM ZnSO_4_) was used as a zinc-sufficient reference condition for comparative analyses in this in vitro system.

AICAR stimulation increased *FoxO1* and *FoxO3* mRNA expression, with a significantly greater induction under zinc-depleted conditions. For *FoxO1* mRNA, this increase tended to be higher in ZnD and lower in Zn20 and Zn40, although the differences were not statistically significant (Figure 4A). In contrast, *FoxO3* mRNA was markedly induced by AICAR in ZnD, whereas this induction was significantly suppressed under zinc-sufficient and zinc-supplemented conditions (Figure 4B). These findings indicate that zinc depletion is associated with greater AMPK-induced transcriptional upregulation of FoxO transcription factors, particularly *FoxO3*, while adequate zinc availability attenuates this response.

### 3.5. AICAR Suppressed S6 Kinase 1 Phosphorylation to a Similar Extent Across All Zinc Conditions

**Figure 5:**
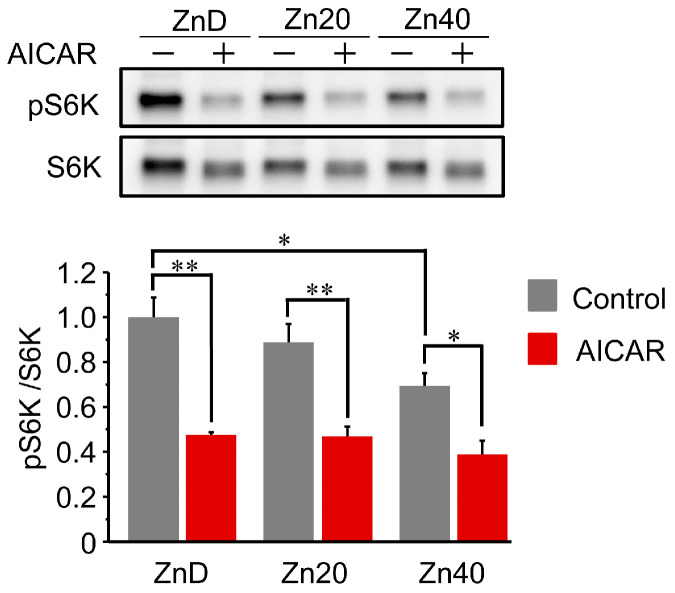
AICAR suppressed S6 kinase 1 phosphorylation to a similar extent across all zinc conditions.Representative Western blot and quantitative analysis of phosphorylated and total p70 S6 kinase 1 (pS6K1 and S6K1) in C2C12 myotubes cultured under ZnD, Zn20, or Zn40 conditions, with or without AICAR treatment. Data are presented as mean ± SEM (n = 3 in each group). * *p* < 0.05, ** *p* < 0.01. Statistical comparisons include Control vs. AICAR within each zinc condition, as well as comparisons among zinc conditions where indicated. Zn20 (20 μM ZnSO_4_) was used as a zinc-sufficient reference condition for comparative analyses in this in vitro system.

At baseline, phosphorylation of p70 S6 kinase 1 (pS6K1/S6K1 ratio) was comparable between ZnD and Zn20, but was significantly lower in Zn40 (Figure 5). Following AICAR treatment, phosphorylation levels were markedly reduced in all groups and reached similar values irrespective of zinc status. These results indicate that AMPK-induced suppression of S6K1 phosphorylation occurs to a comparable extent across zinc conditions, suggesting that zinc availability does not markedly influence the inhibition of the mTORC1-S6K1 anabolic pathway by AMPK activation.

## 4. Discussion

**Figure 6:**
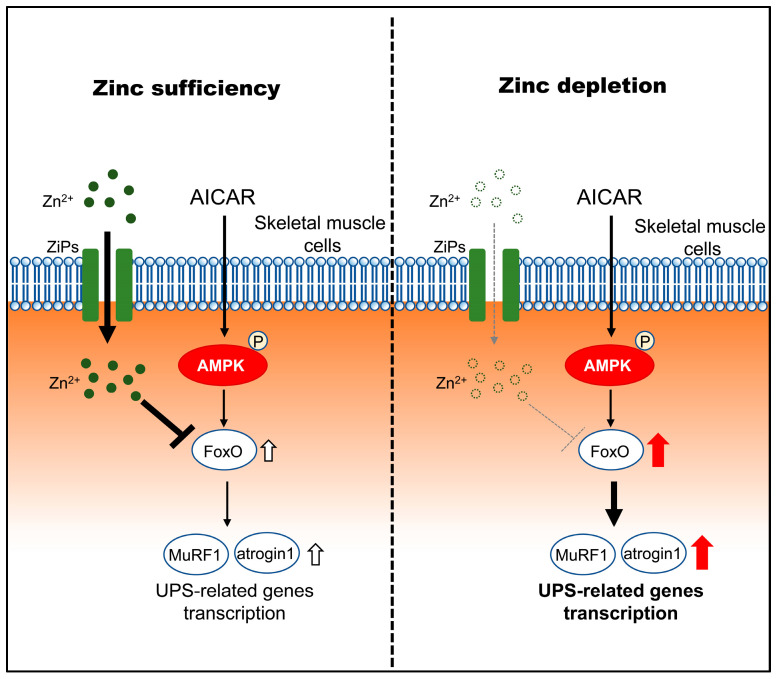
Proposed model illustrating how zinc status modulates downstream atrophic responses to AMPK activation in C2C12 myotubes. Under zinc-sufficient conditions, AMPK activation by AICAR is associated with transcriptional regulation of FoxO and UPS-related genes and is accompanied by increased intracellular zinc availability, potentially involving stress-responsive ZIP transporters (ZIP3, ZIP10, and ZIP14), which may contribute to limiting excessive atrophic gene induction. Under zinc-depleted conditions, AMPK activation fails to increase intracellular zinc availability despite ZIP transcriptional responses and is associated with enhanced transcriptional upregulation of FoxO1/3 and the atrophy-related genes MuRF1 and Atrogin-1, consistent with increased susceptibility to AMPK-induced myotube atrophy.

This study shows that zinc depletion increases susceptibility to AMPK-induced atrophic responses in skeletal muscle cells, characterized by enhanced transcriptional upregulation of FoxO and ubiquitin–proteasome system (UPS)-related genes, without direct evidence of accelerated proteolytic flux, rather than through altered inhibition of the mTORC1-S6K1 pathway. A schematic model summarizing these proposed relationships is shown in Figure 6. Although AICAR increased AMPK phosphorylation similarly across zinc conditions, zinc-depleted myotubes exhibited greater atrophy and higher expression levels of *FoxO1/3*, *MuRF1*, and *Atrogin-1*. These findings indicate that zinc status modifies downstream catabolic responses to AMPK signaling, suggesting that zinc acts not only as a structural or catalytic cofactor but also as a metabolic signal that fine-tunes energy-stress responses. Under zinc-sufficient conditions, AMPK activation is associated with controlled induction of atrophy-related genes, whereas under zinc depletion, this response becomes exaggerated, leading to increased susceptibility to myotube atrophy.

In the present study, short-term (27 h) zinc depletion alone did not induce apparent myotube atrophy or markedly alter the expression of proteolytic markers. However, under AICAR stimulation, zinc depletion is associated with greater AMPK-induced transcriptional upregulation of atrophy-related genes, accelerating myotube atrophy. While prolonged dietary zinc deficiency has been shown to activate the ubiquitin–proteasome system (UPS) and disrupt mitochondrial function in rat skeletal muscle [17], the short-term zinc depletion applied here was likely insufficient to trigger such catabolic responses by itself. Nevertheless, when combined with AMPK-mediated metabolic stress, this condition became detrimental, amplifying transcriptional programs associated with muscle atrophy.

Regarding the relationship between zinc and protein synthesis, previous reports have suggested that zinc promotes protein synthesis in several tissues, including muscle [23,24,25]. Furthermore, zinc has been reported to activate the mTORC1-S6K1 axis [26]. However, the present results showed that zinc depletion did not reduce S6K1 phosphorylation either at baseline or after AMPK activation, suggesting that the anabolic mTORC1-S6K1 branch was largely unaffected. Previous reports in skeletal muscle demonstrated that zinc can activate Akt and, to a lesser extent, mTOR phosphorylation, yet consistent stimulation of the downstream translational effector S6K1 has not been observed [27]. Thus, in skeletal muscle, zinc appears to play a greater role in restraining excessive catabolic gene induction rather than strongly promoting protein synthesis.

The key observation of this study is that AMPK activation increased intracellular zinc concentrations under zinc-sufficient conditions (Zn20 and Zn40), whereas such an increase was not observed under zinc-depleted conditions (ZnD). This finding suggests that AMPK activation elevates the intracellular demand for zinc; however, when extracellular zinc is limited, this demand cannot be met, potentially leading to a failure of the adaptive zinc-dependent modulation of AMPK response. It should be noted that the intracellular zinc values in Figure 2A are intended to demonstrate relative changes across conditions in cultured myotubes and are not meant to represent absolute physiological zinc concentrations in vivo.

Two-way ANOVA revealed distinct regulatory patterns among ZIP transporters (*Zip1*, *Zip3*, *Zip10*, and *Zip14*), which are localized to the plasma membrane and play critical roles in zinc influx [28]. *Zip1* exhibited significant main and interaction effects, showing lower basal expression in Zn40 and an AICAR-induced decrease under ZnD and Zn20, indicating that basal zinc uptake is suppressed under high-zinc or energy-stressed conditions. Because ZIP1 is known as a constitutive importer responsible for basal zinc uptake [28], its downregulation likely reflects feedback suppression during elevated zinc availability or energetic stress.

*Zip3* responded robustly to AICAR across all zinc states, suggesting that it serves as a broadly inducible importer to sustain zinc influx during metabolic stress. This is consistent with previous findings that ZIP3 transcription is responsive to oxidative stress, prolactin stimulation, and changes in zinc availability [29,30,31]. *Zip14* displayed a similar upward trend but reached statistical significance only in ZnD and Zn40, possibly due to higher variability in Zn20 despite a comparable mean increase. ZIP14 has been reported to be induced by inflammatory and metabolic stress–related stimuli, contributing to zinc redistribution under catabolic conditions [32,33].

In contrast, *Zip10* exhibited significant main effects of zinc condition and AICAR treatment, as well as a significant interaction between them, being significantly upregulated by AICAR only in Zn40. These results indicate that AMPK orchestrates a multi-tiered zinc regulatory network in which ZIP1 downregulation limits basal uptake, ZIP3 and ZIP14 provide a general stress-responsive route, and ZIP10 acts as a zinc-dependent, context-specific importer.

*Zip10* showed zinc-dependent, stress-linked behavior: elevated at baseline in ZnD and decreasing with zinc, with an AMPK-induced rise detected only in Zn40. We interpret this as a compensatory importer that supports AMPK-driven zinc demand when extracellular zinc is sufficient but fails to restore cytosolic Zn^2+^ under scarcity. This regulatory mode resembles that of the intestinal transporter ZIP4, which is transcriptionally induced during zinc depletion but functionally downregulated once zinc becomes sufficient through internalization and degradation [34]. Although the precise role of ZIP10 in skeletal muscle remains unclear, its expression increases during C2C12 differentiation [35], and studies in other cell types have demonstrated that ZIP10 plays an important role in zinc influx [36,37]. Thus, ZIP10 may participate in the AMPK-governed adaptive zinc network, serving as a stress-inducible transporter that contributes to intracellular zinc homeostasis.

From a physiological viewpoint, the interplay between AMPK and zinc appears crucial for maintaining the balance between energy conservation and protein turnover. Under sufficient zinc, AMPK activation during exercise or fasting transiently limits protein synthesis but avoids sustained degradation, thereby preserving muscle integrity. In contrast, zinc depletion disrupts this balance, attenuating zinc-dependent modulation of AMPK responses and enhancing atrophic susceptibility. Clinically, suboptimal zinc intake is common among elderly populations and in patients with chronic inflammatory or metabolic diseases such as diabetes and cancer cachexia [38,39,40]. In these conditions, impaired zinc homeostasis may exacerbate AMPK-driven catabolism, accelerating muscle loss. However, extrapolation to clinical settings requires validation in in vivo models.

This study has several limitations. First, because Chelex-100 can chelate divalent cations other than Zn, we did not directly quantify extracellular Mg^2+^, Ca^2+^, or other divalent cations in the final media after Chelex treatment. Therefore, although all groups were prepared using the same Chelex-treated basal medium and only Zn was selectively repleted, we cannot completely exclude potential contributions of altered non-zinc cation availability. Second, although AMPK can activate both the ubiquitin–proteasome and autophagy–lysosome pathways, we observed no detectable change in LC3 expression under any zinc or AICAR condition, suggesting that autophagy contributed little to the observed atrophy. A possible explanation is that the short duration of zinc depletion and AICAR stimulation preferentially engaged transcriptional upregulation of FoxO/UPS-related genes, whereas detectable changes in autophagy markers may require different time courses, stronger stress, or assessment of autophagic flux rather than LC3 abundance alone. Third, the expression of zinc transporters was analyzed only at the mRNA level. Because transcriptional changes do not always reflect functional protein activity, further studies are needed to determine whether the observed changes in ZIP1, ZIP3, ZIP10, and ZIP14 are accompanied by corresponding alterations in protein expression or zinc influx. Fourth, as the present experiments were performed using differentiated C2C12 myotubes in vitro, additional in vivo studies under zinc-depleted conditions will be required to confirm the physiological relevance of these findings. Despite these limitations, the current data provide conceptual insight into how zinc availability modulates downstream atrophic responses to AMPK activation in skeletal muscle cells.

## 5. Conclusions

Zinc depletion increases susceptibility to AMPK-induced atrophic responses in skeletal muscle cells in association with enhanced transcriptional upregulation of FoxO1/3 and UPS-related genes, while leaving AMPK activation and mTORC1-S6K1 inhibition largely unaffected. This imbalance may be related to altered zinc handling under zinc-depleted conditions, potentially involving stress-responsive ZIP transporters such as ZIP3, ZIP10, and ZIP14. Adequate zinc availability therefore permits AMPK signaling to operate in an adaptive manner, limiting excessive atrophic gene induction during metabolic stress. These findings identify zinc status as a key modifier of AMPK-dependent muscle responses and underscore the importance of micronutrient sufficiency in maintaining skeletal muscle integrity.

## Data Availability

The original contributions presented in this study are included in the article/Supplementary Material. Further inquiries can be directed to the corresponding author.

## Supplementary Materials

The following supporting information can be downloaded at: https://www.mdpi.com/article/10.3390/pathophysiology33010012/s1, Table S1: Primer sequences used for Real-time PCR. Figure S1: Original Western blot images.

## Abbreviations

The following abbreviations are used in this manuscript:

AMPK	AMP-activated protein kinase
AICAR	5-aminoimidazole-4-carboxamide ribonucleotide
Atrogin-1	F-box protein 32 (Fbxo32)
DMEM	Dulbecco’s Modified Eagle Medium
FBS	fetal bovine serum
FoxO	forkhead box O
HS	horse serum
LC3	microtubule-associated protein 1 light chain 3
mTORC1	mechanistic target of rapamycin complex 1
MuRF1	muscle RING-finger protein 1 (Trim63)
PBS	phosphate-buffered saline
pS6K1	phosphorylated p70 S6 kinase 1
UPS	ubiquitin–proteasome system
ZIP	Zrt/Irt-like protein (zinc importer)
ZnD	zinc-depleted condition
Zn20	zinc-sufficient condition (20 μM ZnSO_4_)
Zn40	zinc-supplemented condition (40 μM ZnSO_4_)

## References

[1] Cruz-Jentoft A.J., Bahat G., Bauer J., Boirie Y., Bruyere O., Cederholm T., Cooper C., Landi F., Rolland Y., Sayer A.A. (2019). Sarcopenia: Revised European consensus on definition and diagnosis. Age Ageing.

[2] Shafiee G., Keshtkar A., Soltani A., Ahadi Z., Larijani B., Heshmat R. (2017). Prevalence of sarcopenia in the world: A systematic review and meta- analysis of general population studies. J. Diabetes Metab. Disord..

[3] Baracos V.E., Martin L., Korc M., Guttridge D.C., Fearon K.C.H. (2018). Cancer-associated cachexia. Nat. Rev. Dis. Primers.

[4] Hardie D.G., Ross F.A., Hawley S.A. (2012). AMPK: A nutrient and energy sensor that maintains energy homeostasis. Nat. Rev. Mol. Cell Biol..

[5] Kjobsted R., Hingst J.R., Fentz J., Foretz M., Sanz M.N., Pehmoller C., Shum M., Marette A., Mounier R., Treebak J.T. (2018). AMPK in skeletal muscle function and metabolism. FASEB J..

[6] Mounier R., Lantier L., Leclerc J., Sotiropoulos A., Pende M., Daegelen D., Sakamoto K., Foretz M., Viollet B. (2009). Important role for AMPKalpha1 in limiting skeletal muscle cell hypertrophy. FASEB J..

[7] Guo Y., Meng J., Tang Y., Wang T., Wei B., Feng R., Gong B., Wang H., Ji G., Lu Z. (2016). AMP-activated kinase alpha2 deficiency protects mice from denervation-induced skeletal muscle atrophy. Arch. Biochem. Biophys..

[8] Hall D.T., Griss T., Ma J.F., Sanchez B.J., Sadek J., Tremblay A.M.K., Mubaid S., Omer A., Ford R.J., Bedard N. (2018). The AMPK agonist 5-aminoimidazole-4-carboxamide ribonucleotide (AICAR), but not metformin, prevents inflammation-associated cachectic muscle wasting. EMBO Mol. Med..

[9] Peralta S., Garcia S., Yin H.Y., Arguello T., Diaz F., Moraes C.T. (2016). Sustained AMPK activation improves muscle function in a mitochondrial myopathy mouse model by promoting muscle fiber regeneration. Hum. Mol. Genet..

[10] Yan Y., Li M., Lin J., Ji Y., Wang K., Yan D., Shen Y., Wang W., Huang Z., Jiang H. (2022). Adenosine monophosphate activated protein kinase contributes to skeletal muscle health through the control of mitochondrial function. Front. Pharmacol..

[11] Tamura Y. (2021). The Role of Zinc Homeostasis in the Prevention of Diabetes Mellitus and Cardiovascular Diseases. J. Atheroscler. Thromb..

[12] King J.C., Shames D.M., Woodhouse L.R. (2000). Zinc homeostasis in humans. J. Nutr..

[13] Prasad A.S. (2013). Discovery of human zinc deficiency: Its impact on human health and disease. Adv. Nutr..

[14] Ohashi K., Nagata Y., Wada E., Zammit P.S., Shiozuka M., Matsuda R. (2015). Zinc promotes proliferation and activation of myogenic cells via the PI3K/Akt and ERK signaling cascade. Exp. Cell Res..

[15] Mnatsakanyan H., Serra R.S.I., Rico P., Salmeron-Sanchez M. (2018). Zinc uptake promotes myoblast differentiation via Zip7 transporter and activation of Akt signalling transduction pathway. Sci. Rep..

[16] Giugliano R., Millward D.J. (1987). The effects of severe zinc deficiency on protein turnover in muscle and thymus. Br. J. Nutr..

[17] Reddy S.S., Addi U.R., Pullakhandam R., Reddy G.B. (2022). Dietary zinc deficiency disrupts skeletal muscle proteostasis and mitochondrial biology in rats. Nutrition.

[18] Chang E., Kim Y. (2019). Vitamin D Ameliorates Fat Accumulation with AMPK/SIRT1 Activity in C2C12 Skeletal Muscle Cells. Nutrients.

[19] Zhao L., Ha J.H., Okla M., Chung S. (2014). Activation of autophagy and AMPK by gamma-tocotrienol suppresses the adipogenesis in human adipose derived stem cells. Mol. Nutr. Food Res..

[20] Chen S., Luo S., Zou B., Xie J., Li J., Zeng Y. (2023). Magnesium Supplementation Stimulates Autophagy to Reduce Lipid Accumulation in Hepatocytes via the AMPK/mTOR Pathway. Biol. Trace Elem. Res..

[21] Wei C.C., Wu K., Gao Y., Zhang L.H., Li D.D., Luo Z. (2017). Magnesium Reduces Hepatic Lipid Accumulation in Yellow Catfish (*Pelteobagrus fulvidraco*) and Modulates Lipogenesis and Lipolysis via PPARA, JAK-STAT, and AMPK Pathways in Hepatocytes. J. Nutr..

[22] Wei C.C., Luo Z., Hogstrand C., Xu Y.H., Wu L.X., Chen G.H., Pan Y.X., Song Y.F. (2018). Zinc reduces hepatic lipid deposition and activates lipophagy via Zn^2+^/MTF-1/PPARalpha and Ca^2+^/CaMKKbeta/AMPK pathways. FASEB J..

[23] Ehara Y., Yamaguchi M. (1996). Zinc stimulates protein synthesis in the femoral-metaphyseal tissues of normal and skeletally unloaded rats. Res. Exp. Med..

[24] Dorup I., Clausen T. (1991). Effects of magnesium and zinc deficiencies on growth and protein synthesis in skeletal muscle and the heart. Br. J. Nutr..

[25] Hicks S.E., Wallwork J.C. (1987). Effect of dietary zinc deficiency on protein synthesis in cell-free systems isolated from rat liver. J. Nutr..

[26] Lynch C.J., Patson B.J., Goodman S.A., Trapolsi D., Kimball S.R. (2001). Zinc stimulates the activity of the insulin- and nutrient-regulated protein kinase mTOR. Am. J. Physiol. Endocrinol. Metab..

[27] Gao J., Lv Z., Li C., Yue Y., Zhao X., Wang F., Guo Y. (2014). Maternal zinc supplementation enhanced skeletal muscle development through increasing protein synthesis and inhibiting protein degradation of their offspring. Biol. Trace Elem. Res..

[28] Kambe T., Tsuji T., Hashimoto A., Itsumura N. (2015). The Physiological, Biochemical, and Molecular Roles of Zinc Transporters in Zinc Homeostasis and Metabolism. Physiol. Rev..

[29] Xiao C., Kong L., Pan X., Zhu Q., Song Z., Everaert N. (2022). High Temperature-Induced Oxidative Stress Affects Systemic Zinc Homeostasis in Broilers by Regulating Zinc Transporters and Metallothionein in the Liver and Jejunum. Oxidative Med. Cell. Longev..

[30] Kelleher S.L., Lonnerdal B. (2005). Zip3 plays a major role in zinc uptake into mammary epithelial cells and is regulated by prolactin. Am. J. Physiol. Cell Physiol..

[31] Chen S.W., Wu K., Lv W.H., Chen F., Song C.C., Luo Z. (2020). Functional Analysis of Two Zinc (Zn) Transporters (ZIP3 and ZIP8) Promoters and Their Distinct Response to MTF1 and RREB1 in the Regulation of Zn Metabolism. Int. J. Mol. Sci..

[32] Wang G., Biswas A.K., Ma W., Kandpal M., Coker C., Grandgenett P.M., Hollingsworth M.A., Jain R., Tanji K., Lopez-Pintado S. (2018). Metastatic cancers promote cachexia through ZIP14 upregulation in skeletal muscle. Nat. Med..

[33] Aydemir T.B., Chang S.M., Guthrie G.J., Maki A.B., Ryu M.S., Karabiyik A., Cousins R.J. (2012). Zinc transporter ZIP14 functions in hepatic zinc, iron and glucose homeostasis during the innate immune response (endotoxemia). PLoS ONE.

[34] Weaver B.P., Dufner-Beattie J., Kambe T., Andrews G.K. (2007). Novel zinc-responsive post-transcriptional mechanisms reciprocally regulate expression of the mouse Slc39a4 and Slc39a5 zinc transporters (Zip4 and Zip5). Biol. Chem..

[35] Paskavitz A.L., Quintana J., Cangussu D., Tavera-Montanez C., Xiao Y., Ortiz-Miranda S., Navea J.G., Padilla-Benavides T. (2018). Differential expression of zinc transporters accompanies the differentiation of C2C12 myoblasts. J. Trace Elem. Med. Biol..

[36] Hojyo S., Miyai T., Fujishiro H., Kawamura M., Yasuda T., Hijikata A., Bin B.H., Irie T., Tanaka J., Atsumi T. (2014). Zinc transporter SLC39A10/ZIP10 controls humoral immunity by modulating B-cell receptor signal strength. Proc. Natl. Acad. Sci. USA.

[37] Nimmanon T., Ziliotto S., Ogle O., Burt A., Gee J.M.W., Andrews G.K., Kille P., Hogstrand C., Maret W., Taylor K.M. (2021). The ZIP6/ZIP10 heteromer is essential for the zinc-mediated trigger of mitosis. Cell. Mol. Life Sci..

[38] Baarz B.R., Rink L. (2022). Rebalancing the unbalanced aged immune system—A special focus on zinc. Ageing Res. Rev..

[39] Ahmad R., Shaju R., Atfi A., Razzaque M.S. (2024). Zinc and Diabetes: A Connection between Micronutrient and Metabolism. Cells.

[40] Kapala A., Folwarski M., Gazi A. (2024). Cross-sectional observational study: Investigation of zinc concentration in white patients with cancer. Nutrition.

