# Supplementary material for: Zinc Depletion Increases Susceptibility to AMPK-Induced Atrophic Responses in C2C12 Myotubes
Source: Pathophysiology. 2026 Feb 2;33(1):12. doi: 10.3390/pathophysiology33010012 (PMC12922060; doi:10.3390/pathophysiology33010012)
Supplement: Supplementary file 1 [file pathophysiology-33-00012-s001.zip › Table S1. Primer sequences used for Real-time PCR.pdf]

Table S1. Primer sequences used for Real-time PCR

Gene name	Primer sequence (5’ → 3’)	
Zip1	forward	AGGTCAGGTGCTAACCATGAA
	reverse	CTGTTCCTTGTAAGCCAGCGT
Zip3	forward	CCATGGTTCACACACAGAGG
	reverse	AGGGTCCCTGAGGTCACTTT
Zip10	forward	TCATCGCCATCGTTTGCATCA
	reverse	CTCTGGTGAAGGGCTGTGAC
Zip14	forward	GTGTCTCACTGATTAACCTGGC
	reverse	AGAGCAGCGTTCCAATGGAC
MuRF-1	forward	TGTGCAAGGAACACGAAG
	reverse	TGAGAGATGATCGTCTGC
Atrogin1	forward	GCAGAGAGTCGGCAAGTC
	reverse	CAGGTCGGTGATCGTGAG
FoxO1	forward	ACATTTCGTCCTCGAACCAGCTCA
	reverse	ATTTCAGACAGACTGGGCAGCGTA
FoxO3	forward	ACAAACGGCTCACTTTGTCCCAGA
	reverse	TCTTGCCCGTGCCTTCATTCT
Gapdh	forward	AGGTCGGTGTGAACGGATTTG
	reverse	TGTAGACCATGTAGTTGAGGTCA
