# Supplementary figures and images for: Zinc Depletion Increases Susceptibility to AMPK-Induced Atrophic Responses in C2C12 Myotubes
Source: Pathophysiology. 2026 Feb 2;33(1):12. doi: 10.3390/pathophysiology33010012 (PMC12922060; doi:10.3390/pathophysiology33010012)

**Figure S1.** Original Western blot images

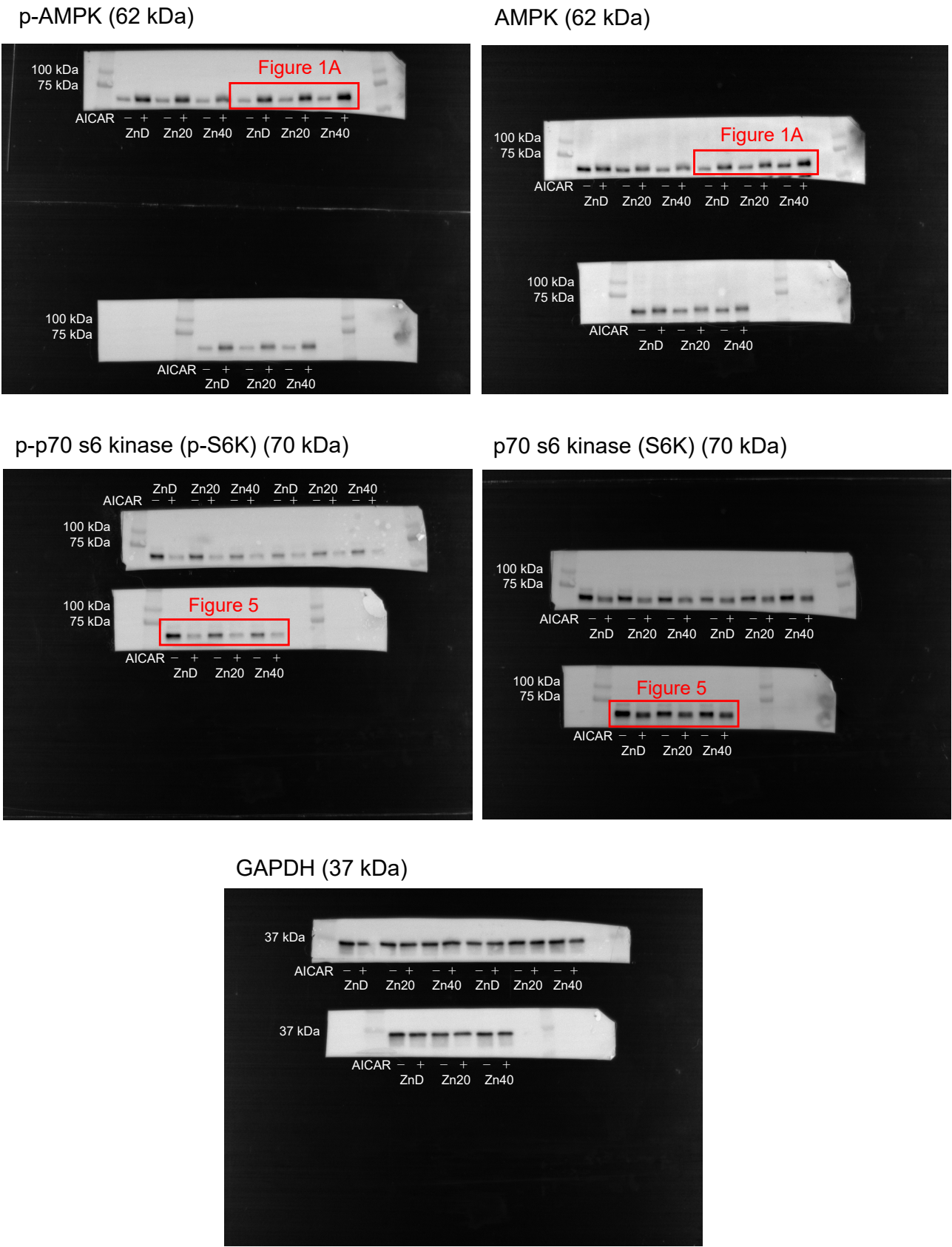

Supplement: Supplementary file 1 [file pathophysiology-33-00012-s001.zip › Figure S1. Original Western blot images.pdf]
